# Ratio of Circulating IFN*γ* ^+^ “Th17 Cells” in Memory Th Cells Is Inversely Correlated with the Titer of Anti-CCP Antibodies in Early-Onset Rheumatoid Arthritis Patients Based on Flow Cytometry Methods of the Human Immunology Project

**DOI:** 10.1155/2016/9694289

**Published:** 2016-05-12

**Authors:** Shigeru Kotake, Yuki Nanke, Toru Yago, Manabu Kawamoto, Tsuyoshi Kobashigawa, Hisashi Yamanaka

**Affiliations:** Institute of Rheumatology, Tokyo Women's Medical University, 10-22 Kawada-cho, Shinjuku, Tokyo 162-0054, Japan

## Abstract

Rheumatoid arthritis (RA) is a systemic autoimmune disease with chronic joint inflammation characterized by activated T cells. IL-17 and Th17 cells play important roles in the pathogenesis of RA. Recently, plasticity in helper T cells has been demonstrated; Th17 cells can convert to Th1 cells. However, it remains to be elucidated whether this conversion occurs in the early phase of RA. Here, we validated the methods of the Human Immunology Project using only the cell-surface marker through measuring the actual expression of IL-17 and IFN*γ*. We also evaluated the expression of CD161 in human Th17 cells. We then tried to identify Th17 cells, IL-17^+^Th17 cells, and IFN*γ* ^+^Th17 cells in the peripheral blood of early-onset RA patients using the standardized method of the Human Immunology Project. Our findings validated the method and the expression of CD161. The ratio of IFN*γ* ^+^Th17 cells in memory T cells was inversely correlated to the titers of anti-CCP antibodies in the early-onset RA patients. These findings suggest that Th17 cells play important roles in the early phase of RA and that anti-IL-17 antibodies should be administered to patients with early phase RA, especially those with high titers of CCP antibodies.

## 1. Introduction

Rheumatoid arthritis (RA) is a systemic autoimmune disease with chronic joint inflammation characterized by activated T cells [1]. We reported in 1999 that IL-17 from activated human T cells in the synovial tissues of patients with RA is a potent stimulator of osteoclastogenesis [2]. Since their identification in 2005 [3], Th17 cells have been reported to play important roles in the pathogenesis of RA [4, 5].

Several findings have supported IL-17 as an important cytokine in the early phase or the disease-onset phase of RA. The peripheral level of IL-17 is significantly high in patients with RA whose disease durations are less than 9 weeks [6]. In addition, the concentration of IL-17 in individuals before disease onset is significantly higher than that in patients after disease onset [7]. In 2013, Chalan et al. reported that circulating CD4^+^CD161^+^T lymphocytes are increased in seropositive arthralgia before the onset of RA but decreased in patients with newly diagnosed RA [8]. In contrast, a regulatory variant in CC chemokine receptor 6 (CCR6), which is a specific marker for Th17 cells distinguishing them from other helper T cells [9, 10], is associated with RA susceptibility [11]. Thus, it is speculated that IL-17 plays an important role in the disease onset or the early phase of RA.

Recently, plasticity in helper T cells has been demonstrated [12]; Th17 cells can convert to Th1 cells [13]. In addition, Th17-cell-derived Th1 cells express CD161, which is a marker of human Th17 cells [14]. Th17-cell-derived Th1 cells producing IFN*γ* have been detected in the synovial fluid from patients with juvenile idiopathic arthritis; thus, these cells are clearly distinct from traditionally known Th1 cells [15–17]. Th17-cell-derived Th1 cells are also named “nonclassic Th1 cells” [18]. In 2013, Chalan et al. reported that CD4^+^CD161^+^T cells in the joints of late-stage RA tend towards a proinflammatory IFN*γ* signature, that is, Th17 cell-derived Th1 or nonclassic Th1 [8]. On the other hand, Th1 rather than Th17 cells were reported to be predominant in the peripheral blood in patients with the late phase of RA whose average disease duration was 13 years [18]. Thus, we hypothesized that Th17 cells convert to Th1 cells during the disease course, even in the early phase of RA.

In 2012, Maecker et al. outlined the state of standardization of flow cytometry assays and summarized the steps that are required for the Human Immunology Project [10]. In the standardization, the definition of particular subsets of immune cells is performed using only cell-surface markers [10]. In the current study, we tried to validate this standardized method on Th17 cells through measuring intracellular IL-17 production. In addition, we also analyzed IFN*γ* ^+^Th17 cells using both the standardized method and the detection of intracellular IFN*γ*. We then evaluated the expression of CD161 in human Th17 cells defined by the standardized method.

In the current study, we evaluated the standardized method of the Human Immunology Project and the expression of CD161 in Th17 cells for the first time. We then tried to identify Th17 cells, Th1 cells, IFN*γ* ^+^Th17 cells, and IL-17^+^Th17 cells in the peripheral blood from early-onset RA patients using both the standardized method and the detection of intracellular cytokines. We found that the standardized method and the expression of CD161 in human Th17 cells were valid and that the ratio of IFN*γ* ^+^Th17 cells in memory Th cells was inversely correlated with the titer of anti-cyclic citrullinated peptide (CCP) antibodies in peripheral blood from early-onset RA patients.

## 2. Patients and Methods

### 2.1. Profiles of Patients

We analyzed 6 patients with early-onset rheumatoid arthritis (RA). The patients met the American College of Rheumatology (ACR) 1987 revised classification criteria. There were 4 females and 2 males. The disease durations were less than 6 months in 5 of 6 patients and that of one patient (#30) was 24 months (Table 1). The RA patients were not treated by DMARDs or corticosteroids when peripheral blood was obtained. Eight osteoarthritis (OA) patients were also analyzed as controls. Ages and genders were not significantly different between the RA patients and OA control patients (data not shown).

The current study was approved by the ethical committee of Tokyo Women's Medical University. Informed consent was obtained from all patients.

### 2.2. Flow Cytometry Analysis for CD4, CD161, and Intracellular IFN-*γ* and IL-17

After separating peripheral blood mononuclear cells (PBMC), memory helper T cells (Th cells) (CD4^+^·CD45RO^+^) were separated using the MACS methods (Memory CD4^+^T Cell Isolation Kit, Miltenyi Biotec). These cells were stimulated with 25 ng/mL PMA (Sigma) and 2 *μ*g/mL ionomycin (Sigma) in the presence of 10 mg/mL brefeldin-A (BFA, Sigma) for 4 h at 37°C in 7% CO_2_. T cells (400 *μ*L) were incubated with 2 mL of 1x FACS lysing solution (Becton Dickinson, Mountain View, CA) for 10 min at room temperature. These cells were washed and incubated with 500 *μ*L of 1x FACS permeabilizing solution (Becton Dickinson) for 10 min at room temperature. These cells were washed again and further incubated with PreCP-Cy5.5-conjugated anti-CD196 (CCR6) antibodies (BD Bioscience), AlexaFluor488-conjugated CD183 (CXCR3) (BD Bioscience), PE-conjugated anti-CD161 antibodies (BD Bioscience), AlexaFluor647-conjugated anti-human IFN*γ* antibodies (BD Bioscience), or AlexaFluo647-conjugated anti-human IL-17 antibodies (BD Bioscience) for 30 min at room temperature in the dark. IgG1k isotype (BD Bioscience) was used as the control. The stained cells were analyzed using FACSCalibur (BD Bioscience).

### 2.3. Statistical Analysis

Data were analyzed using the Wilcoxon test, Spearman's test, and Kruskal-Wallis test (StatView®; Abacus Concepts Inc., Berkeley, CA). Data are presented as the mean ± SD. A significant difference was defined as *p* < 0.05.

## 3. Results

### 3.1. Validation of Human Immunology Project Methods

In the current study, we first confirmed that each parameter was not associated with the patient's age (data not shown). We next tried to validate that Th17 cells, identified as CD183^−^·CD196^+^ cells, in memory CD4^+^T cells according to the methods of the Human Immunology Project [10], actually produce IL-17.

**Figure 1:**
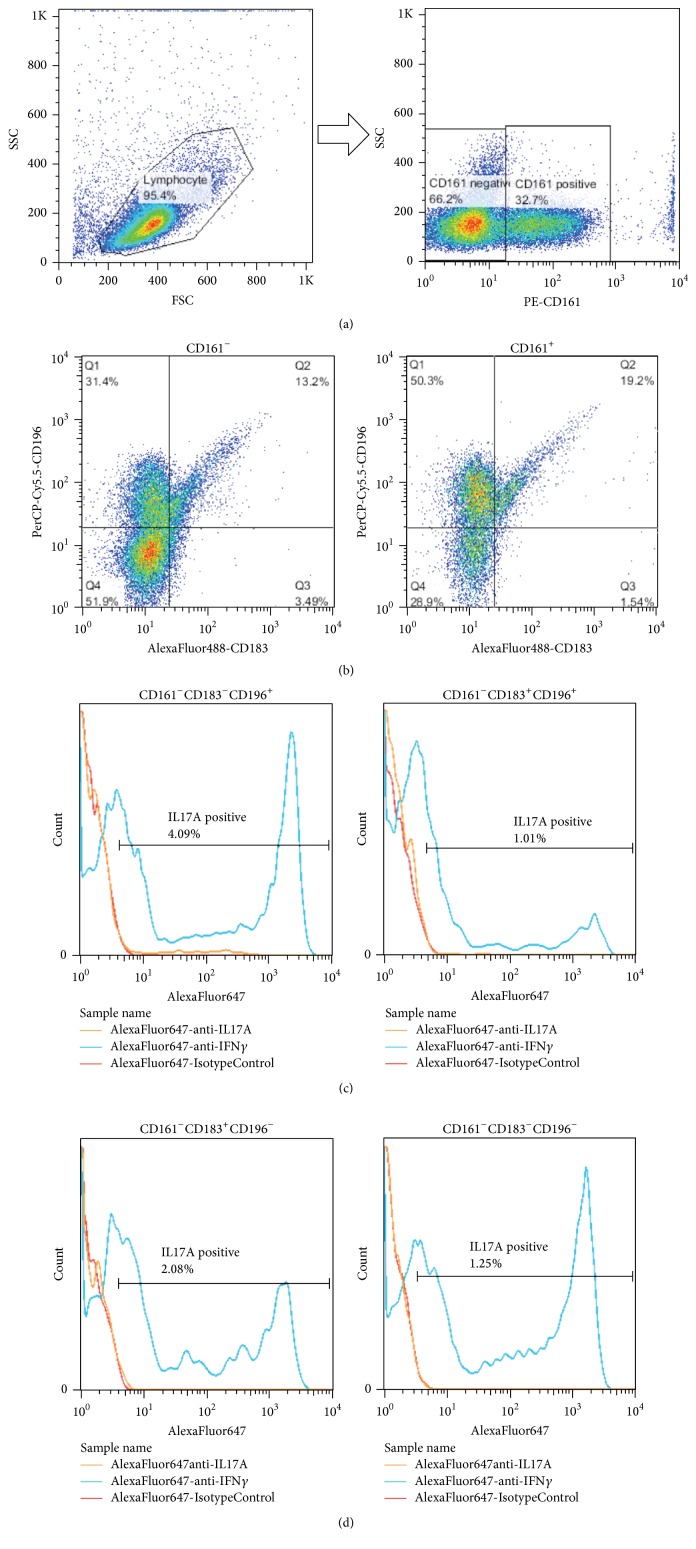
Representative flow cytometry gating and histograms of CD161 negative cells. (a) Separation of CD161 positive cells. (b) Four subsets of CD161 negative or positive cells. (c) Histograms of CD161 negative cells. CD183^−^CD196^+^ cells (Th17) (left) and CD183^+^CD196^+^ cells (right). Orange lines: IL-17; blue lines: IFN*γ*; and red lines: control. (d) Histograms of CD161 negative cells. CD183^+^CD196^−^ cells (Th1) (left) and CD183^−^CD196^−^ cells (right). Orange lines: IL-17; blue lines: IFN*γ*; and red lines: control.

Figure 1 shows the representative data of FCM.  Figure 1(a) shows the separation of CD161 positive cells in FCM gating (Figure 1(a)).  Figure 1(b) shows 4 subsets of CD161 negative cells or positive cells (Figure 1(b)). Figures 1(c) and 1(d) show the histogram of IL-17 and IFN*γ* in the 4 subsets (Figures 1(c) and 1(d)). The ratio of IL-17 detected in each subset was the highest in CD183^−^CD196^+^ cells, that is, Th17 subset (4.09%) (Figure 1(c), left).

**Figure 2:**
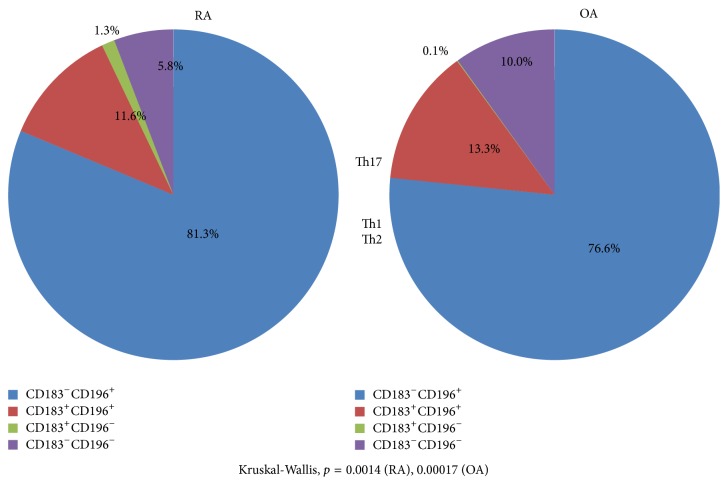
Ratio of IL-17^+^ cells when memory Th cells were divided into 4 subsets according to the positivity of CCR6 (CD196) or CXCR3 (CD183): validation of Human Immunology Project methods.

We analyzed the ratio of IL-17^+^ cells when memory Th cells were divided into 4 subsets according to the positivity of CD183 or CD196 (Figure 2). As shown in Figure 2, 84.3% and 76.6% of IL-17^+^ cells were included in the CD183^−^·CD196^+^ cells in memory CD4^+^T cells in RA and OA, respectively (Kruskal-Wallis, *p* = 0.0014 (RA), 0.00017 (OA)). Thus, the identification of Th17 cells using the Human Immunology Project method was validated.

### 3.2. Validation of CD161 as a Marker of Human Th17 Cells

CD161 has been reported as a marker of human Th17 cells [14]. We next tried to validate that IL-17^+^·CD161^+^ cells are exclusively included in “Th17 cells” identified according to the methods of Human Immunology Project. A representative result is shown in Table 2; 135 of 164 (=135 + 15 + 1 + 13) (82%) IL-17^+^CD161^+^ memory Th cells were included in Th17 cells identified according to the method of the Human Immunology Project (Table 2). In addition, the ratio of CD161^+^ cells in IL-17^+^Th17 cells in RA or OA was 135/135 + 36 (79%) or 107/107 + 29 (79%), respectively (Table 2). These findings suggest that CD161 can be used as a marker of Th17 cells.

### 3.3. The Ratio of Th17 in Memory Th Cells

**Figure 3:**
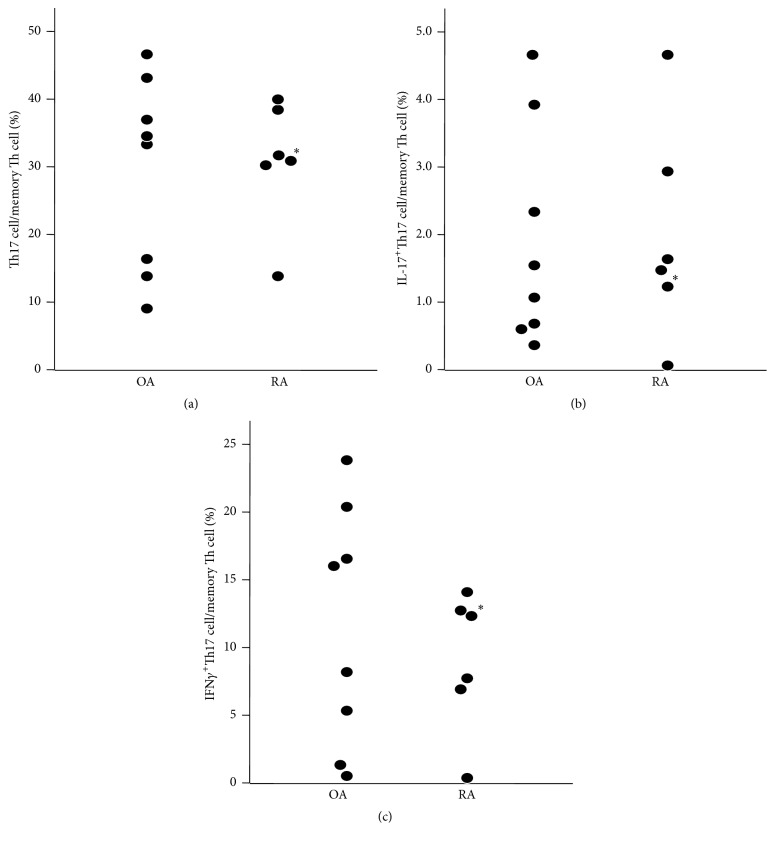
Ratio of Th17 cells in memory Th cells. Th17 cells (a), IL-17^+^Th17 cells (b), and IFN*γ* ^+^Th17 cells (c). *∗*: patient #30 in Table 1.

The ratio of Th17 cells (Figure 3(a)), IL-17^+^Th17 cells (Figure 3(b)) or IFN*γ* ^+^Th17 cells (Figure 3(c)) in memory Th cells was not significantly different between RA and OA.

**Figure 4:**
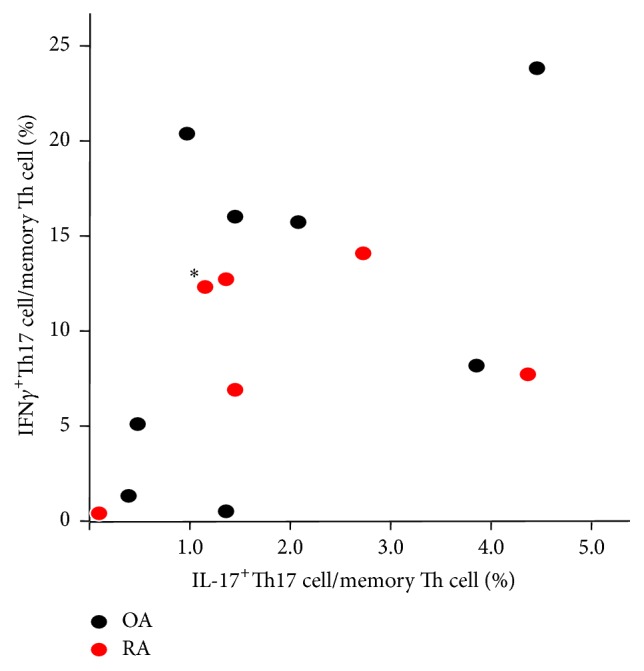
Correlation of the ratio of IL-17^+^Th17 cells and the ratio of IFN*γ* ^+^Th17 cells in memory Th cells. *∗*: patient #30 in Table 1.

The correlation of the ratio of IL-17^+^Th17 cells and the ratio of IFN*γ* ^+^Th17 cells in memory Th cells was not significant in RA or OA (Figure 4).

### 3.4. Correlation of the Ratio of Th Cells and Disease Duration

**Figure 5:**
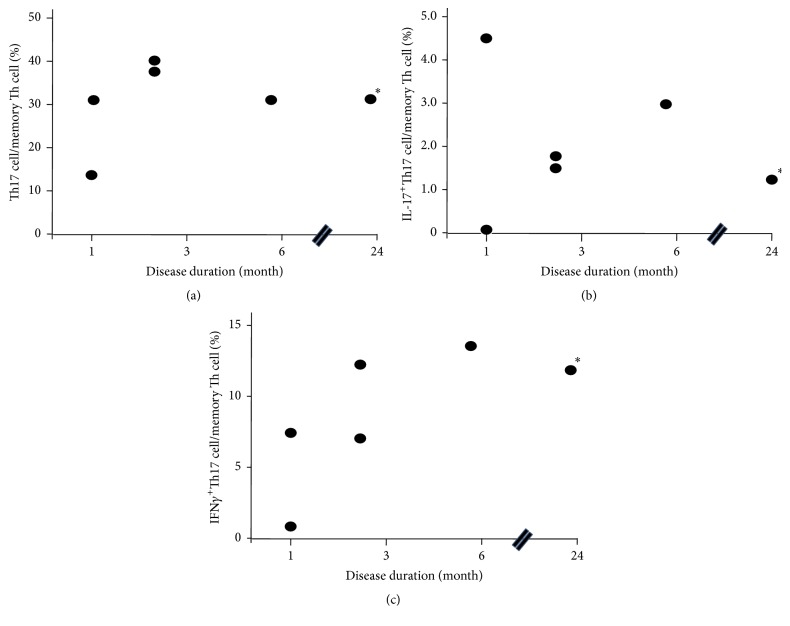
Correlation of the ratio of Th cells and disease duration. Th17 cells (a), IL-17^+^Th17 cells (b), and IFN*γ* ^+^Th17 (c). *∗*: patient #30 in Table 1.

The ratio of Th17 cells and the ratio of IL-17^+^Th17 cells did not correlate with the disease duration (Figures 5(a) and 5(b)). In contrast, the ratio of IFN*γ* ^+^Th17 cells showed a tendency of positive correlation with disease duration, although this did not reach statistical significance (Figure 5(c)).

### 3.5. The Correlation of the Ratio of Th Cells and the Titer of Anti-CCP Antibodies

**Figure 6:**
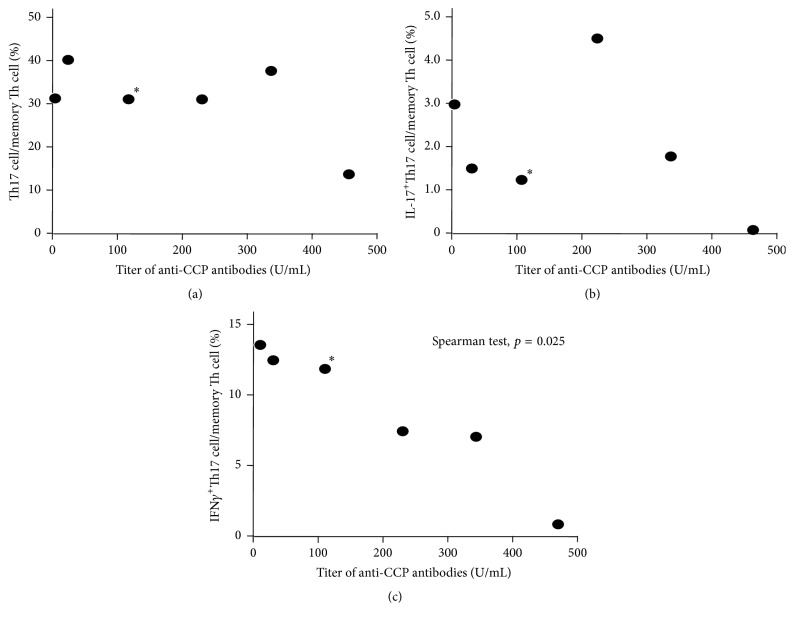
Correlation of the ratio of Th cells and the titer of anti-CCP antibodies. Th17 cells (a), IL-17^+^Th17 cells (b), and IFN*γ* ^+^Th17 (c). *∗*: patient #30 in Table 1.

The ratio of Th17 cells and the ratio of IL-17^+^Th17 cells did not correlate with the titer of anti-CCP antibodies (Figures 6(a) and 6(b)). In contrast, the ratio of IFN*γ* ^+^Th17 cells showed a significant inverse correlation with the titer of anti-CCP antibodies (*p* = 0.025, Figure 6(c)).

## 4. Discussion

We clearly demonstrated that the standardization method of the Human Immunology Project is a valid flow cytometry method for the evaluation of Th17 cells and that CD161 can be used as a marker of human Th17 cells. In addition, we showed that the ratio of IFN*γ* ^+^Th17 cells in memory Th cells was inversely correlated with the titer of anti-CCP antibodies in the peripheral blood from early-onset RA patients.

In the current study, we analyzed IFN*γ* ^+^Th17 cells. Th17 cells were identified by the expression of surface markers according to the Human Immunology Project [10], but not by the production of IL-17. Annunziato et al. reported that human Th17 cells shift to Th17·Th1 cells (Th cells producing both IL-17 and IFN*γ*), after which Th17·Th1 cells shift to “nonclassic Th1 cells” [17]. They named CD161^+^Th1 cells (i.e., Th17 cell-derived Th1 cells) “nonclassic Th1 cells” [17]. It is possible that our IFN*γ* ^+^Th17 cells consist of both Th17·Th1 cells and “nonclassic Th1 cells” because we did not measure the expression of IL-17 in IFN*γ* ^+^Th17 cells.

It has been reported that circulating Th17 cells and Th17·Th1 cells are negatively correlated with anti-CCP titers in early RA patients with disease durations of less than 6 months [19]. In the current study, we showed that the ratio of IFN*γ* ^+^Th17 cells in memory Th cells was negatively correlated with anti-CCP titers in early-onset RA patients; however, Th17 cells or IL-17^+^Th17 cells did not show a significant negative correlation with anti-CCP titers (Figures 6(a), 6(b), and 6(c)). As discussed in the previous paragraph, it is possible that our IFN*γ* ^+^Th17 cells consist of both Th17·Th1 cells and “nonclassic Th1 cells.” The reason for the discrepancy on Th17 cells remains unclear; however, it may depend on variations in the patients analyzed or the methods.

In August 2015, Burmester et al. reported that an anti-IL-17 antibody, secukinumab, induced rapid and significant changes from baseline in DAS28-CRP and in ACR20 and ACR50 response rates compared with placebo on biologic-naïve RA patients and that this treatment was much more effective in Caucasians from Russia than in those from the USA or EU [20]. In their study, the disease durations (mean ± SD) were 6.0 ± 7.1 years, that is, the late phase of RA [20]. In the other clinical trials, the subjects were also the late-phase RA patients, 5.9 ± 6.5~13.0 ± 9.0 (years, mean ± SD) [21–23]. As mentioned in Introduction, IL-17 plays an important role in the preonset or early-onset phase of the pathogenesis of RA [6, 7]. Thus, anti-IL-17 antibodies should be administered to biologic-naïve RA patients in the early phase to obtain a more effective response.

Several findings support anti-CCP-positive RA and anti-CCP-negative RA being different disease entities [24]. In a study to explore the efficacy of methotrexate (MTX) in patients with probable RA and the effect of MTX on the development of RA, radiographic progression was demonstrable in anti-CCP-positive patients, but not in anti-CCP-negative patients [25]. In addition, the presence of anti-CCP-antibodies is a predictive factor for the response to rituximab in RA [26]. Thus, it is suggested that the treatment for RA patients should be approached differently according to the presence or the absence of anti-CCP antibodies.

In the current study, we showed that that the ratio of IFN*γ* ^+^Th17 cells in memory T cells showed lower levels in early RA patients with a high titer of anti-CCP antibodies (Figure 6(c)). The reason of the inverse correlation is not clear. It is speculated that anti-IL-17 antibodies yield an effective response in RA patients with the lower level of IFN*γ* ^+^Th17 cells. As discussed above, the positivity of anti-CCP-antibodies is important in the treatment for RA patients. Taken together, our findings suggest that anti-IL-17 antibodies should be used in early RA patients with a high titer of anti-CCP antibodies to obtain a more effective response to the therapy using anti-IL-17 antibodies. Further studies are needed, analyzing both peripheral blood and synovial tissues, since cell populations in synovial tissues may shift inversely to those in peripheral blood in RA [19, 27].

We validated the standardized method for Th17 cells by detection of intracellular IL-17 production. In addition, we also confirmed the expression of CD161 in Th17 cells defined by the standardized method. The Human Immunology Project is an important analogy to the Human Genome Project [10]; however, the functions of immune cells are also important. In addition, the discovery of new markers for immune cells, such as CD161 for human Th17 cells, should continue. We recommend that both the standardization method using only cell-surface markers and the detection of cytokines and new markers should be performed simultaneously in future studies of the immunological pathogenesis of human RA.

In conclusion, through analyzing the peripheral blood of early-onset RA patients, we demonstrated that the standardized method of Human Immunology Project and the expression of CD161 in human Th17 cells were valid and that the ratio of IFN*γ* ^+^Th17 cells in memory Th cells was inversely correlated with the titer of anti-CCP antibodies in the peripheral blood from early-onset RA patients. These findings suggest that Th17 cells play an important role in the pathogenesis of early phase RA. Anti-IL-17 antibodies should be used in early-phase RA patients with a high titer of anti-CCP antibodies, but not in the late phase of RA or in patients resistant to other biologics, especially anti-TNF antibodies.

## Figures and Tables

**Table 1:** Profile of patients.

Patient number	Sex	Age (year)	Diagnosis 2010 ACR/EULAR	Disease duration (m)	Anti-CCP U/mL (<4.5)	RF IU/mL (<15)	CRP mg/dL	Treatment after the analysis
7	M	47	RA	1	238	37	0.09	MTX 6 mg/w
15	F	71	RA	3	350	50	5.18	Bu
23	F	53	RA	1	469	381	1.18	MTX 6 mg/w
30^*∗*^	M	64	RA	24	115	13	0.30	—
33	F	75	RA	6	<0.6	6	0.08	MTX 6 mg/w
35	F	49	RA	3	19.6	11	0.07	—

^*∗*^Patient #30 had a longer disease duration than the other patients.

MTX: methotrexate; Bu: bucillamine.

The levels in parentheses show the normal ranges of anti-CCP and RF.

**Table 2:** Mean number of IL-17^+^CD161^+^ cells or IL-17^+^CD161^−^ cells (per 10^4^ cells) in 4 portions classified according to the positivity of CD183 or CD196.

		RA	OA
Th17	CD183^−^·CD196^+^		
	IL17A^+^·CD161^+^	135	107
	IL17A^+^·CD161^−^	36	29
	CD183^+^·CD196^+^		
	IL17A^+^·CD161^+^	15	24
	IL17A^+^·CD161^−^	4	3

Th1	CD183^+^·CD196^−^		
	IL17A^+^·CD161^+^	1	0
	IL17A^+^·CD161^−^	1	0

Th2	CD183^−^·CD196^−^		
	IL17A^+^·CD161^+^	13	8
	IL17A^+^·CD161^−^	8	5
